# 

**Published:** 1877-10

**Authors:** 


### CONTENTS:

ARTICLE I.—Annual Session of American Dental Convention, Southern Dental Association and Maryland and District of Columbia Dental Society, in Joint Session ........ 241

ARTICLE II.—Scientific Theories vs. Observed Facts ........ 257

ARTICLE III—North Carolina State Dental Association ........ 262

ARTICLE IV.—Mechanical Dentistry By Dr. C. B. Rohland ........ 262

ARTICLE V.—The Microscope—Its Misinterpretations ........ 271

ARTICLE VI.—Action of Nitrite of Amyl By Dr. W. D. Lane ........ 275

### EDITORIAL, ETC.

Prof. James B. Hodgkin, D. D. S. ........ 276
A New Dental Quarterly—A New Degree in Dentistry ........ 278
A New Method of Pivoting Teeth ........ 280
Editorial Briefs ........ 281
Medical Progress ........ 282
Inhibition of Publication and its Result ........ 282

### BIBLIOGRAPHICAL.

An Index of Diseases and their Treatment ........ 283
The Physicians Visiting List for 1878 ........ 283
Transactions of the American Dental Association for 1876 ........ 283
Transaction of the Illinois State Dental Society for 1877 ........ 284

### MONTHLY SUMMARY.

The Question of Rest during Menstruation ........ 284
Simple Remedy to Stop Bleeding ........ 285
The Perils of the Cigar ........ 285
Hydrobromic Ether as an Anæsthetic ........ 286
The Percussion of Bones ........ 286
On Ranula ........ 286
Lepoma of Tongue ........ 287

